
JOURNAL INFORMATION
==============================
NLM Title Abbreviation: ScientificWorldJournal
Journal ID: ScientificWorldJournal
NLM Journal ID: 101131163
Journal ID: TSWJ

The Scientific World Journal

ISSN: 2356-6140
EISSN: 1537-744X
Publisher: Wiley

ARTICLE INFORMATION
==============================
PMCID: PMC5823221
PMID: 19468652
DOI: 10.1100/tsw.2009.44
Article ID: 657359
Article version: 1
Subjects: Clinical Images

“A Forgotten Disease”: A Case of Lemierre Syndrome

Velagapudi P.
Turagam Mohit *
Are C.
Patel H.
Yekkirala L.
Department of Internal Medicine, University of Illinois, Urbana-Champaign, IL, USA
*Mohit Turagam: turagam2@illinois.eduAcademic Editor: Daniel Batlle

Publication date: 2009
Electronic publication date: 2009 May 20
Volume: 9
First page: 331
Last page: 332
Received 2009 Apr 1; Revised 2009 May 8; Accepted 2009 May 11
Copyright: Copyright © 2009 P. Velagapudi et al.
Copyright year: 2009
License: This is an open access article distributed under the Creative Commons Attribution License, which permits unrestricted use, distribution, and reproduction in any medium, provided the original work is properly cited.
License URL: https://creativecommons.org/licenses/by/3.0/

Keywords: Lemierre’s disease, Fusobacterium necrophorum, internal jugular vein thrombosis

==============================
Clinical Image
*TheScientificWorldJOURNAL* (2009) 9, 331–332
ISSN 1537-744X; DOI 10.1100/tsw.2009.44

TheScientificWorldJOURNAL
www.thescientificworld.com

### "A Forgotten Disease": A Case of Lemierre Syndrome

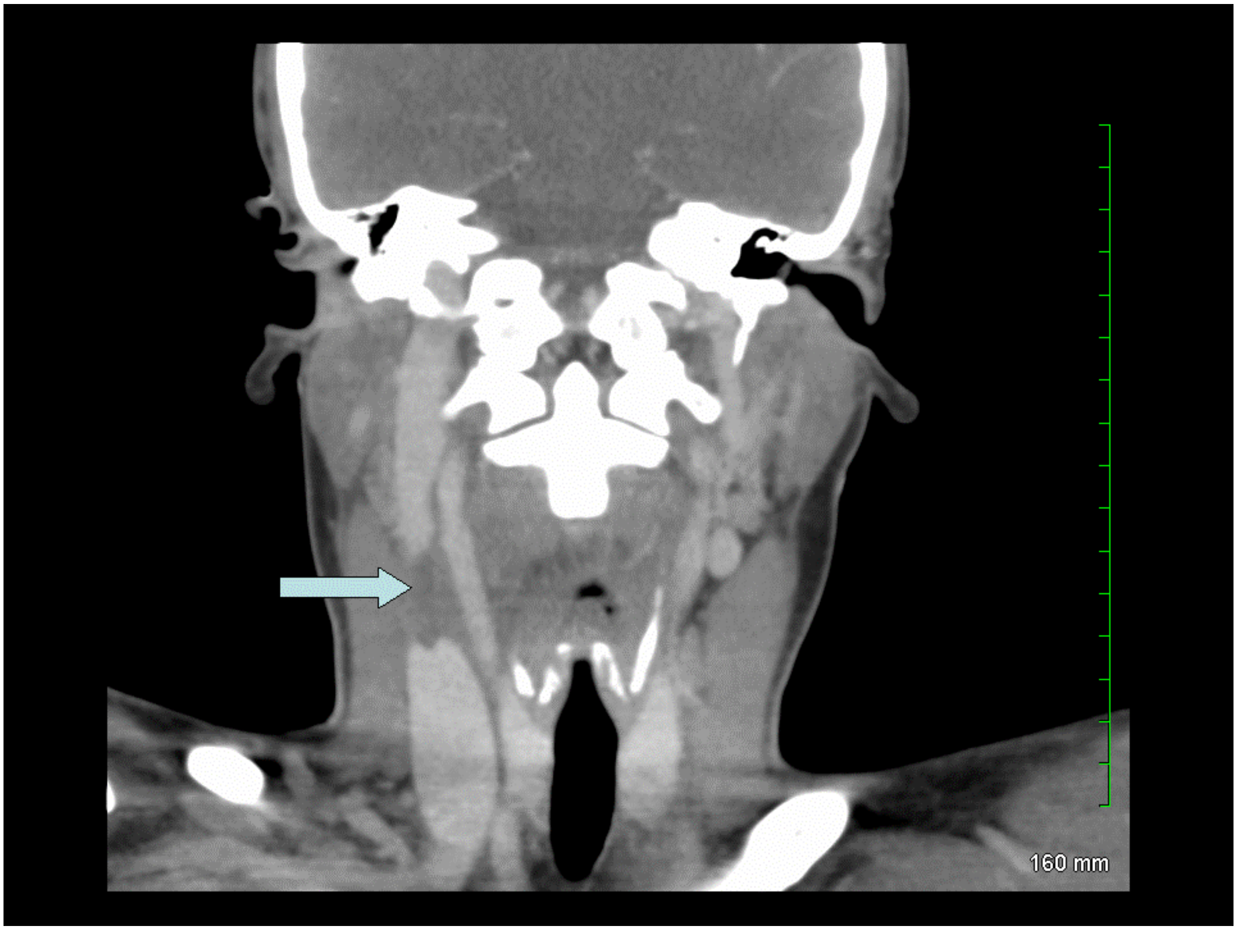

**FIGURE.** CT of the neck with contrast: nonocclusive soft tissue filling defect extending over 2 cm is consistent with a thrombus seen within the right internal jugular vein at the level of the hyoid bone (arrow).

P. Velagapudi, M. Turagam*, C. Are, H. Patel, and L. Yekkirala
*Department of Internal Medicine, University of Illinois, Urbana-Champaign*

**E-mail**: turagam2@illinois.edu

*Received April 1, 2009; Revised May 8, 2009; Accepted May 11, 2009; Published May 20, 2009*

**KEYWORDS**: Lemierre's disease, *Fusobacterium necrophorum*, internal jugular vein thrombosis

*Corresponding author.
©2009 with author.
Published by TheScientificWorld; www.thescientificworld.com

Lemierre's syndrome is a rare, but life-threatening, condition that affects young healthy individuals. It was first described by Dr. Andre Lemierre in 1936. Incidence rates are between 0.6 and 2.3/million populations[1]. It is found more commonly in males, with a male to female ratio of approximately 2:1. Its pathogenesis consists of the development of infectious thrombophlebitis in the internal jugular vein or one of its branches caused by a focal sepsis, mostly localized in the oropharynx, leading to generalized multiorgan metastatic infections, generally to the lung[2,5]. *Fusobacterium necrophorum* and *F. nucleatum* are the two species most often associated with this condition. *Fusobacterium* is a commensal of the normal flora of the human oropharynx, digestive, genital, and urinary tracts, and normally does not invade the mucosa. The exact mechanism of invasion and penetration of the pharyngeal mucosa has not been determined, but the role of the underlying synergistic infectious process with a concomitant decline in host resistance is hypothesized to play an important role[3,4].Unlike other anaerobic bacteria, *Fusobacterium* produces a lipopolysaccharide endotoxin, as well as hemagglutinins, leukocidin, and hemolysin, assisting in the destruction of red and white blood cells[6]; so once invasion of the internal jugular vein is achieved, the resultant bacteremia triggers platelet aggregation and thrombus formation. There have been recent reports of Lemierre syndrome in patients with variations in Toll-like 5 receptor (TLR 5), Tissue factor 603 (TF603), and Plasminogen-Activator-Inhibitor-1 (PAI-1) 4G-4G gene receptors associated with thrombotic events, indicating a possible genetic predisposition associated with the disease[7].

This computerized tomography (CT) of the neck with intravenous contrast is from a 24-year-old female who presented with a 1-day history of fever, hypotension, and respiratory failure. A review of systems revealed a history of sore throat for a week, which resolved 2 days later without any medication. The physical exam was positive for diminished breath sounds bilaterally on lung exam. Complete blood count revealed a leukocytosis of 16,200/µl with 70% neutrophils and 9% bands, hemoglobin of 13.4 mg/dl, and severe thrombocytopenia with a platelet count of 34,000/µl. Comprehensive metabolic panel revealed sodium 140 mmol/l, potassium 2.9 mmol/l, bicarbonate 26 mmol/l, blood urea nitrogen (BUN) 16 mg/dl, creatinine 0.8 mg/dl, calcium 7.2 mg/dl, albumin 2.4 g/dl, total bilurubin 3.1 mg/dl, AST 81 IU/l, ALK 101 IU/l, and ALT 35 IU/l. CT of the chest revealed multiple cavitary opacities in both lungs. Blood cultures were positive for *F. necrophorum*. CT scan of the neck showed a filling defect of the right internal jugular vein consistent with a thrombus and multiple enlarged cervical lymph nodes. Treatment is medical with intravenous antibiotics and anticoagulation.

### REFERENCES

1. Carlson, E.R., Bergamo, D.F., and Coccia, C.T. (1994) Lemierre's syndrome: two cases of a forgotten disease. *J. Oral Maxillofac. Surg*. **52,** 74–78.
2. Moore-Gillon, J., Lee, T.H., Eykyn, S.J., and Phillips, I. (1984) Necrobacillosis: a forgotten disease. *BMJ* **288,** 1526–1527.
3. Jones, C., Siva, T.M., Seymour, F.K., and O'Reilly, B.J. (2006) Lemierre's syndrome presenting with peritonsillar abscess and VIth cranial nerve palsy. *J. Laryngol. Otol.* **120,** 502–504.
4. Mohammed Iqbal Syed et al. (2007) Lemierre syndrome: two cases and a review. *Laryngoscope* **117,** 1605–1610,.
5. Vohra, A., Saiz, E., and Ratzan, K.R. (1997) A young woman with a sore throat, septicaemia, and respiratory failure. *Lancet* **350,** 928.
6. Singhal, A. and Kerstein, M. (2001) Lemierre's syndrome. *South. Med. J.* **94(9),** 886–887.
7. Constantin, J.M., Mira, J.P., and Guerin, R. (2006) Lemierre's syndrome and genetic polymorphisms: a case report. *BMC Infect. Dis*. **6,** 115.

**This article should be cited as follows:**

Velagapudi, P., Turagam, M., Are, C., Patel, H., and Yakkirala, L. (2009) "A forgotten disease": a case of Lemierre syndrome. *TheScientificWorldJOURNAL* **9**, 331–332. DOI 10.1100/tsw.2009.44.
